# Inflammatory Pathways as Promising Targets to Increase Chemotherapy Response in Bladder Cancer

**DOI:** 10.1155/2012/528690

**Published:** 2012-07-02

**Authors:** Zhaowei Zhu, Zhoujun Shen, Chen Xu

**Affiliations:** ^1^Department of Urology, Ruijin Hospital, Shanghai Jiao Tong University School of Medicine, Shanghai 200025, China; ^2^Department of Histology and Embryology, Shanghai Jiao Tong University School of Medicine, Shanghai 200025, China

## Abstract

While more and more physicians are choosing chemotherapy for patients with bladder cancer, the current treatment is still far from satisfactory due to low response rate and severe side effects. Emerging evidence indicates that inflammatory microenvironment is involved in the pathogenesis of bladder cancer. Recent studies have also provided ample evidence that chemotherapy response is influenced by activation of major inflammatory mediators, including transcription factors, cytokines, chemokines, and COX-2. We reviewed all published literature addressing the roles of inflammatory microenvironment in bladder cancer and evaluating emerging evidence that inflammatory pathways represent potential therapeutic targets to enhance chemotherapy of bladder cancer.

## 1. Introduction

Bladder cancer is the second most common urological malignancy in the United States of America and is by far the most frequent urological malignancy in China. This cancer is characterized by high recurrence in the aggressive forms of the disease. Based on incidence and mortality data from several agencies, the American Cancer Society estimated that 70,530 new cancer cases and 14,680 deaths from bladder cancer occurred in the United States in 2010 [1]. In patients with non-muscle-invasive bladder cancer, intravesical instillation of chemotherapy is recommended for reducing the risk of tumor recurrence [2]. Besides, cisplatin-containing perioperative chemotherapy has been explored to improve the treatment outcomes since the 1980s.

Inflammation is regarded as a “secret killer”, and an inflammatory component is present in the microenvironment of most neoplastic tissues, including some in which a direct causal relationship with inflammation has not yet proven. Microbial infections (e.g., *Helicobacter pylori *for gastric cancer and mucosal lymphoma), viral infections (e.g., hepatitis B or C virus for hepatocellular carcinoma), autoimmune disease (e.g., inflammatory bowel disease for colon cancer), and inflammatory conditions of unknown region (e.g., prostatitis for prostate cancer) are recognized as triggers of chronic inflammation which are related to cancer development [3].

**Figure 1:**
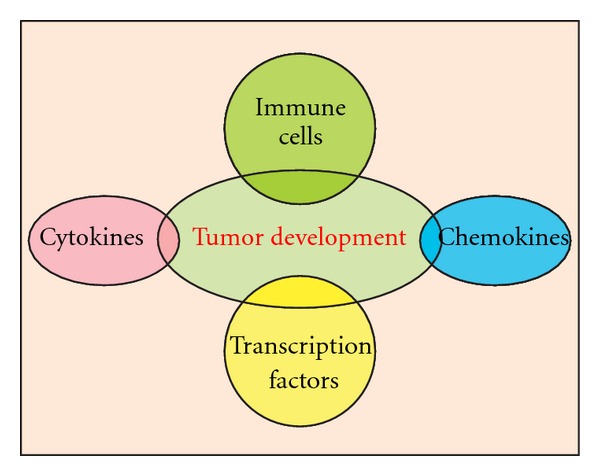
Major components of inflammatory microenvironment in bladder cancer.

Emerging evidence indicates that inflammation plays important roles at different stages of tumor development, including initiation, promotion, invasion, and metastasis [4]. Various lines of evidence have also been reviewed that the chemokine system and cytokines, orchestrated by transcription factors, are key players of cancer-related inflammation (CRI) [5–8] (Figure 1). Inflammation can be considered an enabling characteristic for its contributions to the acquisition of core hallmark capabilities [9]. Furthermore, smouldering inflammation in the tumor microenvironment influences treatment responses to hormones and chemotherapeutic agents. Thus, CRI potentially represents a target for innovative therapeutic strategies [10].

**Figure 2:**
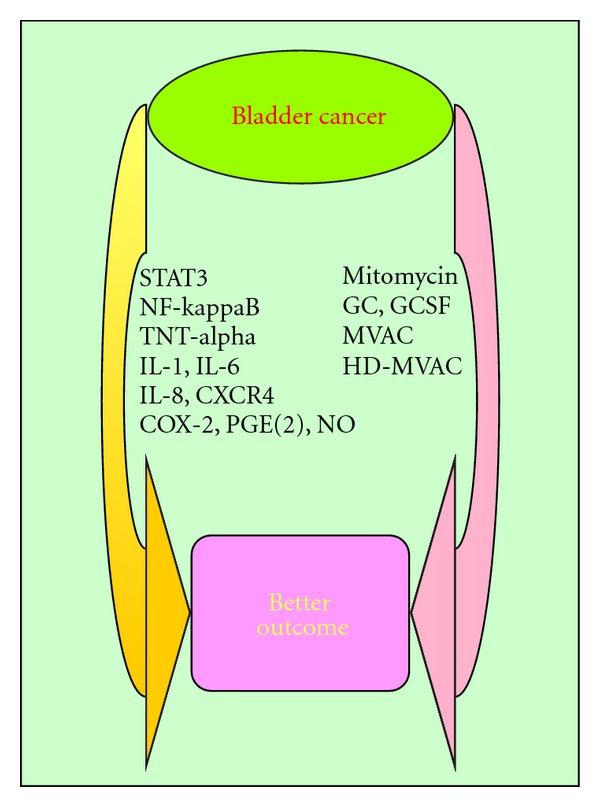
Modulating inflammatory pathways potentially enhance chemotherapy of bladder cancer.

Inflammatory microenvironment may also play critical roles in bladder carcinogenesis and cancer disease progression. Muscle-invasive bladder cancer, particularly invasive squamous cell carcinoma, is directly associated with the presence of chronic urinary tract infection. The identification of transcription factors and their gene products, including cytokines, chemokines, and cyclooxygenase-2 (COX-2), has intensively provided us new clues for novel therapeutic strategies. Considering the functions of inflammatory microenvironment in bladder cancer, they represent potential drug targets to improve the efficacy of chemotherapy agents (Figure 2). In this paper, we summarize the current status of chemotherapy and focus primarily on the CRI in bladder cancer. We will provide ample evidence to show that inflammatory pathways represent promising targets for increasing chemotherapy response in bladder cancer.

## 2. Current Chemotherapy for Bladder Cancer

Transurethral resection (TUR) in noninvasive papillary bladder carcinoma (Ta) and subepithelial invasion (T1) contributes to correct diagnosis and complete resection of visible lesions. In a meta-analysis of seven randomized clinical trials, Sylvester et al. showed that one instillation of chemotherapy immediately after TUR significantly reduced the risk of recurrence after TUR in patients with stage Ta T1-single and -multiple bladder cancer [11]. However, a minimum of one year of intravesical immunotherapy or further instillations of chemotherapy should be administered for patients with an intermediate or high risk of recurrence and an intermediate risk of progression [2]. In a combined analysis comparing intravesical chemotherapy with TUR alone, Pawinski and colleagues demonstrated that chemotherapy prevented recurrence but not progression [12]. Moreover, the 5-year overall recurrence-free rate was only 48.4% in patients treated with intravesical mitomycin C for nonmuscle-invasive bladder cancer [13].

For patients with clinically operable transitional cell carcinoma (TCC) of the urinary bladder, chemotherapy can be delivered before the planned definitive surgery (or radiation). By analyzing two consecutive trials of a Nordic collaborative group, Sherif and colleagues showed that neoadjuvant platinum-based combination chemotherapy was associated with an 8% overall survival advantage [14]. Thus, it has been recommended that neoadjuvant cisplatin-containing combination chemotherapy should be considered and discussed in cases of muscle-invasive, node-negative, and nonmetastatic (N0 M0) urinary bladder carcinoma, irrespective of definitive treatment [15]. Despite the improved survival in patients with locally advanced bladder cancer, neoadjuvant chemotherapy also has potential disadvantages. Although cisplatin is the most effective single agent for the treatment of muscle-invasive bladder cancer, up to 50% of patients are ineligible for cisplatin-containing chemotherapy [15]. Furthermore, Martinez-Pineiro et al.  observed that bladder cancer progressed in 9.7% of patients during chemotherapy [16].

In patients with locally advanced or metastatic TCC of the urothelium treated with gemcitabine/cisplatin (GC) or methotrexate, vinblastine, doxorubicin, and cisplatin (MVAC), long-term overall and progression-free survival were similar [17]. Sternberg and colleagues also observed a benefit in progression-free survival and overall response with high-dose-intensity chemotherapy with MVAC (HD-MVAC) plus granulocyte colony-stimulating factor (GCSF) [18]. Therefore, the first-line treatment for fit patients remains cisplatin-containing combination chemotherapy with GC or MVAC, preferably with GCSF or HD-MVAC with GCSF [15]. However, many studies have reported a high incidence of severe complications from chemotherapy, including granulocytopenia, gastrointestinal toxicity, anemia, thrombocytopenia, and neutropenia [19, 20]. Furthermore, in a large, multicenter, randomized trial, the response rate for GC and MVAC was only 49% and 46%, respectively [19]. Thus, the chemotherapy of bladder cancer is far from satisfactory.

## 3. Immune Cells in Bladder Cancer

### 3.1. Tumor-Associated Macrophages (TAMs)

Macrophages are one of the key inflammatory components of the stroma of human bladder cancer. Functional interactions could probably occur between bladder cancer cells and infiltrating macrophages and affect tumor cell survival and invasion during bladder cancer progression. Dufresne et al. showed that proinflammatory macrophages promoted cellular invasiveness and increased phosphoinositide 3-kinase (PI3-K)/Akt signaling pathway activity in T24 cells [21]. Using an orthotopic urinary bladder cancer model, Yang et al. found massive infiltration of TAM in primary tumor and lymphatic metastasis in lymph nodes. Besides, TAM flocked near lymphatic vessels and express high levels of VEGF-C/D. Due to high expression of VEGFR-3 in lymphatic vascular endothelial cells, TAM could assist lymphangiogenesis by paracrine manner in bladder tumor. Furthermore, the depletion of TAM with clodronate liposome markedly inhibited lymphangiogenesis and lymphatic metastasis [22]. Macrophage infiltration was also observed in COX-2 induced transitional cell hyperplasia (TCH), which was strongly associated with inflammation [23]. Considering higher TAM density in COX-2 high-expression tumor fields, COX-2 may be involved in the process of angiogenesis through increasing TAM infiltration [24].

 It has been suggested that the TAM count was significantly higher in invasive bladder cancers. Patients with a high TAM count showed significantly higher rates of cystectomy, distant metastasis, vascular invasion, and lower survival rate, indicating the prognostic value of TAM count [25, 26]. In patients receiving bacillus Calmette-Guerin (BCG) therapy, high TAM infiltration was significantly associated with worse treatment response and recurrence-free survival [27, 28]. Endothelial Per-Arnt-Sim domain protein 1 (EPAS1), expressed in TAMs, is induced under hypoxia and plays important roles in tumor angiogenesis. Onita et al. identified a higher level of focal TAM infiltration in EPAS1-positive cases than EPAS-1-negative cases [29]. EPAS1 expressing TAMs were found to be associated with a poor prognosis of invasive bladder cancer, suggesting that EPAS1 expressed in a subset of TAMs mediates bladder cancer progression [30].

### 3.2. Myeloid-Derived Suppressor Cells

Whereas TAMs represent the major population of inflammatory cells infiltrating tumors, certain studies suggest that myeloid-derived suppressor cells (MDSC) are also involved in the tumor promotion and metastasis. Initially observed in the late 1970s, MDSC mobilized in many pathological conditions (e.g., inflammatory diseases, trauma, and tumors) and could suppress the immune system. MDSC derive from common progenitors in the bone marrow and accumulate preferentially in blood and spleen with some being recruited directly to the tumor site [31]. Recent years have seen a resurgence of interest in MDSC due to their role in tumor progression and their potential to limit therapeutic responses [32]. It has been demonstrated that the recruitment and function of MDSC are regulated by several cytokines, chemokines, and transcription factors (e.g., VEGF, IL-6, IL-10, CCR-2, and STAT3).

 In view that bladder cancer was associated with enhanced inflammation, Eruslanov et al. examined prostaglandin E metabolism and myeloid cell subsets that infiltrate tumor tissue using two xenograft models of human bladder cancer. As expected, human bladder tumors secreted substantial amounts of Prostaglandin E. They also found that fast growing SW780 bladder tumor xenografts were infiltrated with heterogeneous CD11b myeloid cell subsets including TAM and MDSC. Based on these observations, they proposed that enhanced cancer-related inflammation and deregulated PGE metabolism in tumor microenvironment promote immunosuppressive protumoral phenotype of myeloid cells in bladder cancer [33]. Furthermore, Eruslanov et al. found that bladder tumors were infiltrated with monocyte-macrophage CD11b(+) HLA-DR(+) and granulocytic CD11b(+) CD15(+) HLA-DR(−) myeloid cells. They demonstrated that these highly activated inflammatory myeloid cells represented a source of multiple chemokines/cytokines and might contribute to inflammation and immune dysfunction in bladder cancer [34].

Immunogene therapy was administered to animals with large malignant mesothelioma and lung cancer tumors followed by three weekly administrations of Cisplatin/Gemcitabine, which were commonly used to treat these tumors in clinical practice. This regimen resulted in markedly increased antitumor efficacy through multiple immune-mediated mechanisms, including decreases in immunosuppressive cells (e.g., MDSC) [35]. Clinical trials indicate that GC is the standard of care for patients with locally advanced or metastatic urothelial TCC. Therefore, MDSC represent potential therapeutic target to improve chemotherapy of bladder cancer.

### 3.3. T Cells

T cells are one of the most frequently found immune cells within the tumor microenvironment. Based on the T-cell receptors they express, mature T cells have historically been characterized into two major groups: *γδ* and *αβ*. T cells are further classified according to their effector functions as CD8+ cytotoxic T cells (CTLs) and CD4+ helper T (Th) cells, which include Th1, Th2, Th17, and T regulatory (Treg) cells, as well as natural killer T (NKT) cells [4]. Of note, many of the T-cell subsets may be also involved in promotion, progression, and metastasis of bladder carcinoma.

 Increasing evidence indicates that T cells can exert both tumor-suppressive and -promoting effects in bladder cancer. In mouse subcutaneous and lung metastasis bladder cancer models, Zhang et al. found that a novel GM-CSF surface modified vaccine significantly inhibited tumor growth and extended survival. Interestingly, more CTLs and Th cells were observed at tumor sites and in peripheral blood in the novel therapeutic vaccine treated group than in other control groups [36]. Naoe et al. found that the BCG-pulsed DCs could activate the NKT cells and *γδ*T cells, which showed unspecific cytotoxic activity against a bladder cancer cell line [37]. Using the genetically modified bladder cancer MBT2 cells, Furukawa also demonstrated that the antitumor activity of IL-21 was mediated through the activation of CTLs in vivo [38].

 Agarwal et al. observed that bladder cancer patients developed Th2 dominant status with deficient type 1 immune response which showed tendency to reversal following therapy [39]. Satyam et al. also demonstrated that in bladder tumor patients a marked polarization existed towards the expression of Th2 type cytokines while Th1 remained suppressed [40]. Helicobacter pylori protein HP-NAP, a toll-like receptor (TLR) 2 ligand, could drive the differentiation of Th 1 cells both in vitro and in vivo by creating an IL-12-enriched milieu. In a mouse model of bladder cancer implant, local administration of HP-NAP decreases tumor growth by triggering tumor necrosis. Moreover, the effect was accompanied by a significant accumulation of both CD4+ and CD8+ IFN-*γ*-secreting cells, within tumor and regional lymph nodes [41]. What determines the same T-cell subset antitumorigenic or protumorigenic remains largely unknown and may hold the key to the development of more successful treatment.

 Th17 cells were found to be enriched in the tumors of patients with bladder cancer compared with the peripheral blood of patients and controls, whereas a higher proportion of Treg cells were observed in peripheral blood compared with healthy controls. Furthermore, IL-2 could convert tumor-infiltrating Treg cells into Th17 cells and downregulate suppressive capacity of Treg cells. As a first study to define the role of Th17 cells in bladder cancer, Chi et al. suggested that the balance between Th17 and Treg cells might be also involved in the development or progression of bladder cancer, providing a rationale for investigating novel therapeutic modalities for patients with invasive disease [42].

## 4. Cytokines in Bladder Cancer

As a critical mediator of inflammation, tumor necrosis factor (TNF) represents one of the potential molecular links between chronic inflammation and cancer. Initially considered as a cytokine resulting in hemorrhagic necrosis of experimental cancers, TNF was later found to exert protumoral functions. Moreover, TNF-alpha could afford protection to chemotherapeutic agents through NF-kappaB-mediated antagonism of apoptosis signaling [43]. Feng et al.  observed that increased TNF-alpha expression was noticed in tumorous tissue compared with healthy urothelium. Besides, they found expressional change of TNF-alpha was associated with angiogenesis of bladder tumor, especially in bladder cancer development [44]. In human bladder cancer cells, TNF-alpha could stimulate the secretion of matrix metalloproteinase-9 (MMP9) which has been implicated in tumor invasion and metastasis [45]. Besides, Lee and colleagues showed that TNF-alpha-induced invasion, migration, and proliferation and MMP9 expression could be suppressed by cordycepin [46].

As a major pleiotropic cytokine in tumor-host interactions, Interleukin (IL)-1 represents a family of two agonistic proteins, IL-1 alpha and IL-1 beta. Using TaqMan probe quantitative RT-PCR technique, Seddighzadeh et al. found that the levels of IL-1alpha mRNA expression did not differ significantly between tumors of different grade or stage. However, low tumoral IL-1alpha expression predicted decreased survival of patients with poorly differentiated tumors and of patients with invasive tumors. Therefore, they concluded that IL-1alpha was important for bladder cancer biology, and that measurements of this cytokine might contribute to pretreatment characterization of bladder cancer [47]. In an extended patient material, they also found that IL-1alpha was expressed by tumor rather than stromal cells by immunohistochemistry. Furthermore, low levels of IL-1alpha mRNA expression are associated with an increased risk for cancer-specific death in the investigated material [48].

IL-6, a multifunctional cytokine acting on epithelial and immune cells, binds to plasma membrane receptor complexes containing the common signal transducing receptor chain glycoprotein 130, leading to activation of the Janus kinase and mitogen-activated protein kinase pathways. The proliferative and survival effects of IL-6 are mainly mediated by the transcription factor STAT3. IL-6 plays decisive roles in the inflammation, immune response, and hematopoiesis [49], thus therapeutic targeting of IL-6 and its receptor in cancer has strong biologic rationale. The IL-6 variant genotype (C/C) was found to be associated with an increased bladder cancer risk [50]. It has been shown that IL-6 might provide a selective growth advantage to N-methyl-N-nitrosourea-initiated bladder epithelial cells in vitro, and thus accounting for the dramatic enhancement of inflammation-associated rat bladder carcinogenesis. Furthermore, Okamoto et al.  found that the growth of bladder carcinoma cells was significantly inhibited by anti-IL-6 neutralizing antibody or the antisense oligonucleotide, concluding that IL-6 functioned as an autocrine growth factor for bladder carcinoma cells [51].

 To facilitate cancer cell detection and opsonization, IL-6 is produced by immune cells resulting in the release of serum C-reactive protein (CRP) by hepatocytes which, in turn, activate the complement system. CRP levels reflect IL-6 levels and can be easily measured in daily practice. While CRP is mostly known as a sensitive inflammatory marker indicating systemic response to pathogens, its prognostic potential has been recently investigated in a subset of cancers. Ito et al. demonstrated that CRP was an independent prognostic factor for overall survival of patients with castration-resistant prostate cancer treated with docetaxel [52]. Park et al.  reported that preoperative high-sensitivity CRP levels might be independent predictive factors for late recurrence of renal cell carcinoma [53]. Gakis et al. assessed the predictive value of serum CRP in patients with invasive bladder cancer and found that serum CRP was an independent risk factor of cancer-specific survival after radical cystectomy. Moreover, they developed the first predictive model based on serum CRP and standard pathological risk factors (tumor stage, node density, resection margin status, C-reactive protein level), termed TNR-C Score and proved that the addition of serum CRP to these three established pathological risk factors increased their predictive accuracy [54]. In patients with invasive bladder cancer, the prognostic importance of CRP and its possible usefulness for addressing the need of adjuvant treatment warrants further investigation.

## 5. Chemokines and Chemokine Receptors in Bladder Cancer

Chemokines and chemokine receptors, key regulators in inflammation and cancer, play important roles in tumor progression, invasion, and metastasis. Emerging evidence indicates that chemokines are produced by tumor cells as well as by cells of the tumor microenvironment including cancer-associated fibroblasts, mesenchymal stem cells, endothelial cells, TAM, and more recently tumor-associated neutrophils [7]. In different types of cancer, chemokine receptor expression was found to be strongly correlated with organ-specific metastasis. Through blocking chemokines or their receptors, it is possible to decrease the dissemination of cancer cells or inhibit their ability to survive and develop tumors. Thus, chemokine system represents a promising innovative target for disease-directed therapy in human malignancies.

IL-8 and CXCR4 was significantly overexpressed in tumor samples as compared to normal bladder tissue [55]. Urinary IL-8 levels were also significantly elevated in patients with TCC [56]. Inoue K. and colleagues demonstrated that IL-8 enhanced angiogenic activity by induction of MMP9 expression and subsequently regulated the tumorigenicity and metastasis in human bladder cancer [57]. Nishizawa et al. identified that CXCR4 expression was induced in high-grade superficial bladder tumors, including carcinoma in situ and invasive bladder tumors [58]. In view of the crosstalk between phosphoinositide 3-kinases and CXCR4 in chronic lymphocytic leukemia, phosphoinositide 3-kinases inhibitors target CXCR4 signaling and overcome stromal cell-mediated drug resistance [59]. Moreover, blocking agents and antibodies directed against CXCR4 prevent metastasis of different cancers in the preclinical setting [60].

## 6. Mediators of Inflammatory Signaling in Bladder Cancer

### 6.1. COX-2

COX are the key enzymes that convert an array of fatty acid substrates into proinflammatory prostanoids, which promotes tumorigenesis partially by inhibiting apoptosis and by increasing cell proliferation and angiogenesis. As an inducible isoform of COX, the elevated levels of COX-2 have been implicated in a wide variety of malignant diseases, and metabolites produced through the action of COX-2 on arachidonic acid have been found to influence various carcinogenic pathways [61]. Epidemiological results have suggested that use of nonsteroidal anti-inflammatory drugs, prototypic inhibitors of COX-2, is associated with reduced risk of certain malignancies [62–64]. In patients with invasive TCC of the urinary bladder, short-term treatment with celecoxib, a selective COX-2 inhibitor, increased apoptosis in tumor tissues, which justified further study of the antitumor effects of COX-2 inhibitors in invasive TCC [65]. Considering the important role of celecoxib in cancer treatment, recent studies have identified a potential benefit for adding celecoxib to standard advanced colorectal cancer chemotherapy regimens to increase their efficacy [64]. However, COX-2 inhibitors have been associated with an increased cardiovascular risk, and studies are still ongoing regarding these side effects [66].

Klein et al. suggested that high levels of COX-2 were sufficient to cause TCH and carcinomas in the urinary bladder [23]. Moreover, overexpression of COX-2 in bladder cancer was associated with higher pathological stage, lymphovascular invasion, and metastases to lymph nodes [67]. Sabichi et al. did not observe a clinical benefit for celecoxib in preventing recurrence of nonmuscle-invasive bladder cancer in a randomized controlled trial. However, celecoxib had a marginally significant effect on reducing metachronous recurrences compared with placebo [68]. Thus, inhibition of COX-2 should continue to be pursued as a potential chemopreventive and therapeutic strategy. Piroxicam could decrease tumor volume in dogs with naturally occurring invasive TCC of the urinary bladder. When piroxicam was combined with cisplatin chemotherapy, induction of tumor apoptosis and reduction in angiogenic factor concentrations were also observed, indicating that piroxicam/cisplatin had antitumor activity against canine TCC, a disease that closely mimics human invasive urinary bladder cancer [69, 70].

### 6.2. Prostaglandin E(2)

Prostaglandins are lipid compounds which mediate various physiological functions. Prostaglandin E(2) (PGE(2)), whose synthesis is driven by cyclooxygenase enzymes including COX-2, is the most abundant prostanoid in humans. Using a Wistar rat model, Shi et al. found that the level of PGE(2) in bladder TCC was much higher than those in normal bladder and bladder papilloma. Therefore, they concluded that increasing PGE(2) level might play an important role in bladder carcinogenesis [71]. Despite insufficient ability to induce malignant transformation, forced COX-2 expression in immortalized urothelial cells contributed to increased PGE(2) production and increased invasion through Matrigel [72]. Eschwège et al. further showed that PGE synthase was involved in the tumorogenesis of the TCC. Besides, there was a relationship between the arachidonate acid metabolic PGE(2) pathway expression and the aggressiveness of the TCC of the urinary bladder [73]. For patients in whom bladder cancer is in remission, Wheeler et al. also observed a lower level of urinary PGE(2) compared with those who have active disease [74].

### 6.3. Nitric Oxide

Nitric oxide (NO) is formed from L-arginine by NO synthase. As an important signaling molecule, NO regulates a diverse range of physiological and cellular processes. In benign and malignant human bladder tissues, NO exerts several functions through different pathways. Ehsan et al. showed that increased angiogenesis was a cGMP-mediated effect (those resulting from the NO/sGC/cGMP pathway). However, non-cGMP-mediated effects (those resulting from the NO/oxidative pathway) were observed more often in bladder carcinoma [75]. In view that BCG induces NO synthase activity, the BCG effect on bladder cancer may partly be due to stimulation of local NO formation [76, 77]. It has been suggested that nitric oxide-donating NSAIDs (NO-NSAIDs) were safer and more efficient than traditional NSAIDs [78]. NCX 1102, an NO-donating sulindac derivative, demonstrated a greater antiproliferative potency compared to its parent molecule sulindac in three-human urothelial epithelial carcinoma cell lines, indicating that this new NO-NSAID may have therapeutic impact in the management of bladder cancer [79]. Furthermore, another NO-NSAID (NCX 4040) could led to apoptosis via a mitochondrial-dependent mechanism, and thus probably improve treatment efficacy of bladder cancer [80].

## 7. Transcription Factors in Bladder Cancer

Nuclear factor-kappaB (NF-kappaB), which is originally discovered in the kappa chain of immunoglobulin and in nucleus of B cells, is now recognized as a transcription factor which is ubiquitous to all cell types and present in the cytoplasm in its resting stage. As a key orchestrator of innate immunity/inflammation, the presence of constitutively active NF-kappaB has been observed in most tumor cells. Upregulation of NF-kappaB activation has also been reported in both bladder cancer tissue and cisplatin-resistant human bladder cancer cell line [81, 82]. Recent study suggested that ursolic acid could sensitize cancer cells to chemotherapeutic agents by suppression of NF-kappaB [83]. Kamat et al. observed that curcumin potentiated the apoptotic effects of chemotherapeutic agents (gemcitabine and paclitaxel) through downregulation of NF-kappaB and its gene products (e.g., COX-2) in human bladder cancer cells [84]. Thus, it is likely to improve chemotherapy of bladder cancer by targeting NF-kappaB.

Signal transducers and activators of transcription 3 (STAT3),  a point of convergence for oncogenic signaling pathways, is constitutively activated in various tumor cells. STAT3 activation is dependent on a variety of factors, such as IL-6 and IL-17 [85, 86]. Recent studies have provided evidence for the critical role of the NF-kappaB-IL-6-STAT3 cascade. Persistently activated STAT3 was shown to maintain constitutive NF-kappaB activity in both cancer cells and tumor-associated hematopoietic cells, thus providing evidence for the relation between oncogenic signaling pathways within the inflammatory microenvironment [87]. By targeting the STAT3 signaling pathway in bladder cancer cells, Chen et al.  reported that STAT3 was involved in the carcinogenesis of bladder cancer [88]. Furthermore, long-term nicotine exposure-induced chemoresistance is associated with activation of STAT3, and inhibition of STAT3 by siRNA or a specific inhibitor restored chemosensitivity in bladder cancer cells [89]. STAT3 activation was crucial for exhibition of malignant characteristics in T24 bladder cancer cells [90], indicating that STAT3 signaling pathway represents as a potential therapeutic target for bladder cancer.

The normal function of hypoxia-inducible factor-1 (HIF-1) complex (a heterodimer composed of an alpha unit and a beta subunit) is to regulate the transcriptional response to hypoxic stress [91]. Under normal conditions, the alpha subunit is enzymatically hydroxylated, whereas the beta subunit are constitutively expressed [92, 93]. Under conditions of low oxygen tension, HIF-1 alpha is accumulated and stimulates the production of growth factors, including vascular endothelial growth factor (VEGF), epidermal growth factor receptor, and transforming growth factor alpha, which are crucial regulators of tumor angiogenesis, invasion, and metastasis [94]. There are lines of evidence indicating that overexpression of HIF-1 alpha correlates with angiogenesis and unfavourable prognosis in urothelial carcinoma [25, 95–97]. Furthermore, Fechner and colleagues observed that hypoxia led to elevated levels of HIF-1alpha and VEGF and compromised treatment with gemcitabine [98], providing a rational for clinical trials evaluating agents targeting this pathway to enhance chemotherapy of bladder cancer.

 In addition to HIF-1 alpha, extracellular adenosine and adenosi006Ee receptors are also important mediators of the effects of hypoxia in tumor microenvironment. Activated T cells could adapt to the changing energy supplies in hypoxic areas of inflamed tissues by using HIF1 to switch to glycolysis as the main source of energy and by signaling through extracellular-adenosine receptors [99, 100]. Phelps et al. detected the presence of transcripts for the adenosine A1, A2A, and A2B receptors using RT-PCR experiments. Furthermore, application of specific adenosine receptor ligands resulted in concentration-dependent increases in intracellular calcium, and the rank order of potency was typical of adenosine A2B receptors [101]. Of note, intermittent intratumor injection of aminophylline (a nonselective adenosine receptor antagonist) and ATL801 (a selective A2B receptor antagonist) slowed the growth of MB49 bladder tumors in syngeneic mice, suggesting that blockade of adenosine receptor has potential antitumor effects [102].

## 8. Chemotherapy-Induced Inflammation

Of note, strong inflammatory response can be initiated in response to cancer treatment, including surgery, chemotherapy, and radiation. Chemotherapy kills tumor cells primarily through necrosis, a proinflammatory form of cell death [4]. Martins et al. demonstrated that chemotherapeutic agents caused the release of ATP into the extracellular space as they induced tumor cell death [103]. The accumulation of high levels of adenosine in tumors suppresses immune responses through adenosine A2A and A2B receptors signaling [102]. Adenosine production from extracellular ATP has also attracted attention as a mechanism of Treg cells-mediated immune regulation. Deaglio et al. reported that inflammatory ATP was converted to adenosine through the action of CD73 and CD39 which were expressed by Treg cells. Of note, the coordinated expression of CD39/CD73 on Treg cells and the adenosine A2A receptor on activated T effector cells generated immunosuppressive loops, indicating roles in the inhibitory function of Treg cells [104]. Stella et al. described, for the first time, the differential pattern of ectonucleotidases in human bladder cancer cell lines [105]. Clayton et al. demonstrated that exosomes, small vesicles secreted by bladder cancer cells, expressed CD39 and CD73 which suppressed T cells through adenosine production. Interestingly, this T-cell inhibition was mediated through the adenosine 2A receptor [106]. The above studies have revealed evidence of a relationship between purinergic signaling and bladder cancer. Overall before or after chemotherapy purinergic signaling may also play a very important role for antitumor immune responses and disease progression.

 Considering that efficacy of chemotherapy is affected by the host immune system at multiple levels, the net outcome of chemotherapy-induced inflammation is controversial. On the one hand, chemotherapy can promote tumor development by inducing an inflammatory microenvironment [107]. Hence, inhibition of chemotherapy-induced inflammation is likely to enhance the treatment of cancer and provide survival advantage to certain patients with malignancies. On the other hand, chemotherapy stimulates antigen presentation by tumor-infiltrating dendritic cells and induces production of cytokines, leading to enhanced adaptive antitumor immune response [4, 108]. To date, however, little has been known about how chemotherapy-induced inflammation influences treatment response of bladder cancer. Many of the underlying mechanisms remain to be elucidated in future studies.

## 9. Conclusions

The global burden of urinary bladder cancer has aroused wide concern internationally. The diagnosis and treatment for bladder cancer are in a state of evolution, driven largely by advanced instruments and techniques. It has been suggested that chemotherapy represents important modality for patients with bladder cancer. However, the results are not satisfactory due to low response rate and severe side effects. There is mounting evidence that inflammation plays critical roles in the pathogenesis of bladder cancer. Interference with the inflammatory microenvironment has been confirmed to support antitumor activities. It is expected that modulating the inflammatory microenvironment potentially enhance chemotherapy of bladder cancer. To date, however, there are no prospective, comparative, larger-scale clinical trials to combine anti-inflammatory approaches with traditional chemotherapy in bladder cancer. We recommend future trials and preclinical studies to develop innovative regimens which help to increase chemotherapy response of bladder cancer with less adverse effects.
